# Supplementary material for: Genetic and Epigenetic Factors in Ulcerative Colitis: A Narrative Literature Review
Source: Genes (Basel). 2025 Sep 15;16(9):1085. doi: 10.3390/genes16091085 (PMC12470167; doi:10.3390/genes16091085)
Supplement: Supplementary file 1 [file genes-16-01085-s001.zip › Supplementary Material-Table S1.pdf]

***Table S1. Genes involved in Ulcerative Colitis** [64]*

Gene	Description	*Gene*	Description
ABI1	abl interactor 1	*LINC01845*	long intergenic non-protein coding RNA 1845
ACTR1A	actin related protein 1A	*LINC02009*	long intergenic non-protein coding RNA 2009
ADCY7	adenylate cyclase 7	*LINC02132*	long intergenic non-protein coding RNA 2132
ALDH7A1P4	aldehyde dehydrogenase 7 family member A1 pseudogene 4	*LINC02324*	long intergenic non-protein coding RNA 2324
AMZ1	archaelysin family metallopeptidase 1	*LINC02537*	long intergenic non-protein coding RNA 2537
ANKRD12	ankyrin repeat domain 12	*LINC02635*	long intergenic non-protein coding RNA 2635
APEH	acylaminoacyl-peptide hydrolase	*LINC02757*	long intergenic non-protein coding RNA 2757
AQP12B	aquaporin 12B	*LINC02888*	long intergenic non-protein coding RNA 2888
ATG5	autophagy related 5	*LINC02920*	long intergenic non-protein coding RNA 2920
ATXN10	ataxin 10	*LINC02929*	long intergenic non-protein coding RNA 2929
BLTP1	bridge-like lipid transfer protein family member 1	*LINC02937*	long intergenic non-protein coding RNA 2937
BRD2	bromodomain containing 2	*LINC02940*	long intergenic non-protein coding RNA 2940
BSN	bassoon presynaptic cytomatrix protein	*LINC03004*	long intergenic non-protein coding RNA 3004
BTBD3	BTB domain containing 3	*LINC03007*	long intergenic non-protein coding RNA 3007
BTF3L4P3	basic transcription factor 3 like 4 pseudogene 3	*LITAF*	lipopolysaccharide induced TNF factor
BTRC	beta-transducin repeat containing E3 ubiquitin protein ligase	*LNC-LBCS*	lncRNA bladder and prostate cancer suppressor, hnRNPK interacting
C1orf141	chromosome 1 open reading frame 141	*LPP*	LIM domain containing preferred translocation partner in lipoma
C6orf47	chromosome 6 open reading frame 47	*LPXN*	leupaxin
C6orf47-AS1	C6orf47 antisense RNA 1	*LRRC3C*	leucine rich repeat containing 3C
CALM3	calmodulin 3	*LSP1*	lymphocyte specific protein 1
CAMK2A	calcium/calmodulin dependent protein kinase II alpha	*MAML2*	mastermind like transcriptional coactivator 2
CARD9	caspase recruitment domain family member 9	*MAP3K8*	mitogen-activated protein kinase kinase kinase 8
CARINH	colitis associated IRF1 antisense regulator of intestinal homeostasis	*MFSD13A*	major facilitator superfamily domain containing 13A
CASC15	cancer susceptibility 15	*MICB-DT*	MICB divergent transcript
CASP3P1	caspase 3 pseudogene 1	*MIEN1*	migration and invasion enhancer 1
CBX3P9	CBX3 pseudogene 9	*MIR4435-2HG*	MIR4435-2 host gene

Gene	**Description**	*Gene*	**Description**
DSE	dermatan sulfate epimerase	*MIR4679-2*	microRNA 4679-2
CCHCR1	coiled-coil alpha-helical rod protein 1	*MMP24OS*	MMP24 opposite strand
CCL2	C-C motif chemokine ligand 2	*MRPL42P4*	mitochondrial ribosomal protein L42 pseudogene 4
CCL20	C-C motif chemokine ligand 20	*MST1*	macrophage stimulating 1
CCL7	C-C motif chemokine ligand 7	*NCR3*	natural cytotoxicity triggering receptor 3
CCND3P1	cyclin D3 pseudogene 1	*NFKB1*	nuclear factor kappa B subunit 1
CCNL2	cyclin L2	*NOTCH1*	notch receptor 1
CCNY	cyclin Y	*NOTCH4*	notch receptor 4
CCNY-AS1	CCNY antisense RNA 1	*NR5A2*	nuclear receptor subfamily 5 group A member 2
CCR7	C-C motif chemokine receptor 7	*NUS1P4*	NUS1 pseudogene 4
CCRL2	C-C motif chemokine receptor like 2	*NXPE1*	neurexophilin and PC-esterase domain family member 1
CD28	CD28 molecule	*NXPE2P1*	neurexophilin and PC-esterase domain family member 2 pseudogene 1
CD6	CD6 molecule	*NXPE4*	neurexophilin and PC-esterase domain family member 4
CDH3	cadherin 3	*OSMR*	oncostatin M receptor
CDK18	cyclin dependent kinase 18	*OTUD3*	OTU deubiquitinase 3
CDKAL1	CDK5 regulatory subunit associated protein 1 like 1	*PA2G4P2*	proliferation-associated 2G4 pseudogene 2
CEBPA	CCAAT enhancer binding protein alpha	*PARK7*	Parkinsonism associated deglycase
CELSR3	cadherin EGF LAG seven-pass G-type receptor 3	*PDGFB*	platelet derived growth factor subunit B
CEP128	centrosomal protein 128	*PHC2*	polyhomeotic homolog 2
CEP131	centrosomal protein 131	*PHYKPL*	5-phosphohydroxy-L-lysine phospho-lyase
CEP72-DT	CEP72 divergent transcript	*PIGCP2*	phosphatidylinositol glycan anchor biosynthesis class C pseudogene 2
CFB	complement factor B	*PITX1-AS1*	PITX1 antisense RNA 1
CH25H	cholesterol 25-hydroxylase	*PLCG2*	phospholipase C gamma 2
CHORDC1P5	CHORDC1 pseudogene 5	*PLCL1*	phospholipase C like 1 (inactive)
CHP1	calcineurin like EF-hand protein 1	*PLPP3*	phospholipid phosphatase 3
CRTC3	CREB regulated transcription coactivator 3	*PNKD*	PNKD metallo-beta-lactamase domain containing
CTIF	cap binding complex dependent translation initiation factor	*POLR1HASP*	POLR1H antisense, pseudogene
CUL2	cullin 2	*PPIAP34*	peptidylprolyl isomerase A pseudogene 34
CXCR1	C-X-C motif chemokine receptor 1	*PRDM1*	PR/SET domain 1
CXCR2	C-X-C motif chemokine receptor 2	*PRKCB*	protein kinase C beta
CYCSP42	CYCS pseudogene 42	*PROCR*	protein C receptor
CYTH1	cytohesin 1	*PRXL2B*	peroxiredoxin like 2B
DAP	death associated protein	*PSEN2*	presenilin 2
DELEC1	deleted in esophageal cancer 1	*PSMB8*	proteasome 20S subunit beta 8
DLD	dihydrolipoamide dehydrogenase	*PSORS1C1*	psoriasis susceptibility 1 candidate 1
DNAJB6P4	DNAJB6 pseudogene 4	*PTGIR*	prostaglandin I2 receptor

Gene	Description	*Gene*	Description
DNMT3A	DNA methyltransferase 3 alpha	*PTPN2*	protein tyrosine phosphatase non-receptor type 2
DSE	dermatan sulfate epimerase	*PUS10*	pseudouridine synthase 10
DUSP7	dual specificity phosphatase 7	*PVALEF*	parvalbumin like EF-hand containing
EHMT2	euchromatic histone lysine methyltransferase 2	*RGS14*	regulator of G protein signaling 14
EHMT2-AS1	EHMT2 and SLC44A4 antisense RNA 1	*RLN2*	relaxin 2
EIF2S2P3	eukaryotic translation initiation factor 2 subunit 2 beta pseudogene 3	*RN7SL636P*	RNA, 7SL, cytoplasmic 636, pseudogene
ELF1	E74 like ETS transcription factor 1	*RNF186*	ring finger protein 186
EMSY	EMSY transcriptional repressor, BRCA2 interacting	*RNF186-AS1*	RNF186 antisense RNA 1
EPS15P1	epidermal growth factor receptor pathway substrate 15 pseudogene 1	*RNU1-150P*	RNA, U1 small nuclear 150, pseudogene
ERGIC1	endoplasmic reticulum-golgi intermediate compartment 1	*RNU2-14P*	RNA, U2 small nuclear 14, pseudogene
ERRFI1-DT	ERRFI1 divergent transcript	*RNU4ATAC4P*	RNA, U4atac small nuclear 4, pseudogene
FAM238C	family with sequence similarity 238 member C	*RNU6-222P*	RNA, U6 small nuclear 222, pseudogene
FAP	fibroblast activation protein alpha	*RNU6-481P*	RNA, U6 small nuclear 481, pseudogene
FCAR	Fc alpha receptor	*RNU6-793P*	RNA, U6 small nuclear 793, pseudogene
FCGR2A	Fc gamma receptor IIa	*RNU7-147P*	RNA, U7 small nuclear 147 pseudogene
FGFR1OP2P1	FGFR1 oncogene partner 2 pseudogene 1	*RNU7-167P*	RNA, U7 small nuclear 167 pseudogene
GALC	galactosylceramidase	*RORC*	RAR related orphan receptor C
GATD3	glutamine amidotransferase class 1 domain containing 3	*RPL23AP12*	ribosomal protein L23a pseudogene 12
GLI3	GLI family zinc finger 3	*RPL29*	ribosomal protein L29
GLYAT	glycine-N-acyltransferase	*RPL3*	ribosomal protein L3
GNA12	G protein subunit alpha 12	*RPL6P17*	ribosomal protein L6 pseudogene 17
GPR35	G protein-coupled receptor 35	*RPS21P8*	ribosomal protein S21 pseudogene 8
GPR65	G protein-coupled receptor 65	*RPS27P5*	ribosomal protein S27 pseudogene 5
GRB7	growth factor receptor bound protein 7	*RTEL1*	regulator of telomere elongation helicase 1
GSDMA	gasdermin A	*RTEL1-TNFRSF6B*	RTEL1-TNFRSF6B readthrough (NMD candidate)
GSDMB	gasdermin B	*SEMA6D*	semaphorin 6D
HCP5	HLA complex P5	*SEPTIN9*	septin 9
HECTD4	HECT domain E3 ubiquitin protein ligase 4	*SFMBT1*	Scm like with four mbt domains 1
HNRNPA1P41	heterogeneous nuclear ribonucleoprotein A1 pseudogene 41	*SH2B1*	SH2B adaptor protein 1
HORMAD1	HORMA domain containing 1	*SLC34A1*	solute carrier family 34 member 1
HORMAD2	HORMA domain containing 2	*SLC39A11*	solute carrier family 39 member 11
HOXA11-AS	HOXA11 antisense RNA	*SLC7A10*	solute carrier family 7 member 10
HOXA13	homeobox A13	*SLIT1*	slit guidance ligand 1
IDI1P2	IDI1 pseudogene 2	*SMAD3*	SMAD family member 3

Gene	**Description**	*Gene*	**Description**
IFIH1	interferon induced with helicase C domain 1	*SMAD7*	SMAD family member 7
IFNG-AS1	IFNG antisense RNA 1	*SMARCE1*	SWI/SNF related, matrix associated, actin dependent regulator of chromatin, subfamily e, member 1
IKZF3	IKAROS family zinc finger 3	*SMG1P5*	SMG1 pseudogene 5
IL10	interleukin 10	*SMURF1*	SMAD specific E3 ubiquitin protein ligase 1
IL12B	interleukin 12B	*SNAPC4*	small nuclear RNA activating complex polypeptide 4
IL17REL	interleukin 17 receptor E like	*SNRPGP8*	small nuclear ribonucleoprotein polypeptide G pseudogene 8
IL18R1	interleukin 18 receptor 1	*SPMIP7*	SPMIP7
IL1R1	interleukin 1 receptor type 1	*STAT3*	signal transducer and activator of transcription 3
IL1R2	interleukin 1 receptor type 2	*STAT4*	signal transducer and activator of transcription 4
IL2	interleukin 2	*STK24-AS1*	STK24 antisense RNA 1
IL21	interleukin 21	*TAP2*	transporter 2, ATP binding cassette subfamily B member
IL21R	interleukin 21 receptor	*TET2*	tet methylcytosine dioxygenase 2
IL23R	interleukin 23 receptor	*TET2-AS1*	TET2 antisense RNA 1
IL4R	interleukin 4 receptor	*TGFBR3*	transforming growth factor beta receptor 3
IL7R	interleukin 7 receptor	*TMBIM1*	transmembrane BAX inhibitor motif containing 1
INAVA	innate immunity activator	*TMCO4*	transmembrane and coiled-coil domains 4
INHBA-AS1	INHBA antisense RNA 1	*TNFRSF14*	TNF receptor superfamily member 14
INSL4	insulin like 4	*TNFRSF6B*	TNF receptor superfamily member 6b
INTS11	integrator complex subunit 11	*TNFSF15*	TNF superfamily member 15
IPMK	inositol polyphosphate multikinase	*TNRC18*	trinucleotide repeat containing 18
IRF1	interferon regulatory factor 1	*TNXB*	tenascin XB
IRF2BP2	interferon regulatory factor 2 binding protein 2	*TOM1*	target of myb1 membrane trafficking protein
IRF5	interferon regulatory factor 5	*TRAF3IP2*	TRAF3 interacting protein 2
IRGM	immunity related GTPase M	*TRAF3IP2-AS1*	TRAF3IP2 antisense RNA 1
ITGA4	integrin subunit alpha 4	*TRIM15*	tripartite motif containing 15
ITLN1	intelectin 1	*TRIM26*	tripartite motif containing 26
ITLN2	intelectin 2	*TSBP1*	testis expressed basic protein 1
JAK2	Janus kinase 2	*TSBP1-AS1*	TSBP1 and BTNL2 antisense RNA 1
KCP	kielin cysteine rich BMP regulator	*TSEN15P3*	tRNA splicing endonuclease subunit 15 pseudogene 3
KIAA0319	KIAA0319	*TTC33*	tetratricopeptide repeat domain 33
KIR3DL2	killer cell immunoglobulin like receptor, three Ig domains and long cytoplasmic tail 2	*TYK2*	tyrosine kinase 2
KPNA7	karyopherin subunit alpha 7	*U8*	U8 small nucleolar RNA

Gene	Description	*Gene*	Description
LCOR	ligand dependent nuclear receptor corepressor	*UBE2L3*	ubiquitin conjugating enzyme E2 L3
LINC00484	long intergenic non-protein coding RNA 484	*UBE2V2*	ubiquitin conjugating enzyme E2 V2
LINC00511	long intergenic non-protein coding RNA 511	*UQCRHP1*	ubiquinol-cytochrome c reductase hinge protein pseudogene 1
LINC00581	long intergenic non-protein coding RNA 581	*VEGFA*	vascular endothelial growth factor A
LINC00598	long intergenic non-protein coding RNA 598	*Y_RNA*	Y RNA
LINC01082	long intergenic non-protein coding RNA 1082	*YDJC*	YdjC chitooligosaccharide deacetylase homolog
LINC01271	long intergenic non-protein coding RNA 1271	*YES1*	YES proto-oncogene 1, Src family tyrosine kinase
LINC01399	long intergenic non-protein coding RNA 1399	*ZBTB40*	zinc finger and BTB domain containing 40
LINC01475	long intergenic non-protein coding RNA 1475	*ZFP90*	ZFP90 zinc finger protein
LINC01620	long intergenic non-protein coding RNA 1620	*ZGPAT*	zinc finger CCCH-type and G-patch domain containing
LINC01767	long intergenic non-protein coding RNA 1767	*ZPBP2*	zona pellucida binding protein 2
