# Supplementary material for: Genetic and Epigenetic Factors in Ulcerative Colitis: A Narrative Literature Review
Source: Genes (Basel). 2025 Sep 15;16(9):1085. doi: 10.3390/genes16091085 (PMC12470167; doi:10.3390/genes16091085)
Supplement: Supplementary file 1 [file genes-16-01085-s001.zip › Supplementary Material-Table S2.pdf]

Table S2. *Variants and risk allele associated with UC identified by GWAS studies [7, 64, 67-91].*

Variant and risk allele (rs)	P-value	OR	CI	Gene	References
rs11150589-A	3×10^{-10}	1.0831269	[1.06-1.11]	*Y_RNA*	[1] / [79]
rs7657746-A	5×10^{-10}	1.0973365	-	*BLTP1*	
rs113010081-G	9×10^{-10}	1.1354866	[1.09-1.18]	*LINC02009, CCRL2*	
rs1517352-C	2×10^{-9}	1.0808891	-	*STAT4*	
rs4743820-A	4×10^{-9}	1.0842872	-	*LINC02937, LINC00484*	
rs907611-A	1×10^{-8}	1.0787733	[1.05-1.1]	*LSP1*	
rs913678-A	1×10^{-8}	1.0787014	-	*LINC01271, RN7SL636P*	
rs7404095-G	2×10^{-8}	1.0744067	-	*PRKCB*	
rs2382817-A	2×10^{-8}	1.0740336	[1.05-1.1]	*PNKD, TMBIM1*	
rs6426833-?	1×10^{-55}	-	-	*OTUD3, RNF186-AS1*	
rs2836878-G	7×10^{-53}	1.25304	-	*LINC02940, RPL23AP12*	
rs3024505-A	5×10^{-43}	1.2520686	[1.22-1.28]	*Y_RNA, IL10*	
rs1801274-A	1×10^{-41}	1.1863672	-	*FCGR2A*	
rs3806308-G	6×10^{-39}	1.1850245	-	*RNF186-AS1*	
rs3197999-A	2×10^{-37}	1.1865767	[1.16-1.21]	*MST1*	
rs7134472-A	6×10^{-37}	1.1745031	[1.15-1.2]	*IFNG-AS1*	
rs4409764-A	2×10^{-36}	1.1710649	[1.15-1.2]	*LINC01475*	
rs56167332-A	7×10^{-27}	1.1518488	[1.13-1.18]	*IL12B, LINC01845*	
rs12946510-A	1×10^{-25}	1.141072	[1.12-1.17]	*IKZF3, GRB7*	
rs6466198-?	2×10^{-25}	-	-	*DLD, PIGCP2*	
rs10800309-?	4×10^{-25}	-	-	*FCGR2A, RNU6-481P*	
rs4380874-A	6×10^{-25}	1.1398237	[1.11-1.16]	*DLD, PIGCP2*	
rs7608910-G	1×10^{-23}	1.1355296	[1.11-1.16]	*PUS10*	
rs2155219-A	8×10^{-21}	1.1254078	[1.1-1.15]	*EMSY, LINC02757*	
rs661054-?	3×10^{-20}	-	-	*NXPE1*	
rs561722-G	4×10^{-20}	1.1328671	-	*NXPE2P1, NXPE1*	
rs6466198-?	1×10^{-18}	-	-	*DLD, PIGCP2*	
rs2816958-G	1×10^{-18}	1.1982042	-	*NR5A2*	
rs6426833-A	3×10^{-76}	1.2616084	-	*OTUD3, RNF186-AS1*	
rs254560-A	4×10^{-10}	1.0823938	[1.06-1.11]	*PITX1-AS1*	
rs11641184-A	4×10^{-10}	1.0811478	[1.06-1.11]	*LITAF*	
rs1405108-C	6×10^{-10}	1.0854406	[1.06-1.11]	*PLCL1, RNU7-147P*	
rs12103-A	1×10^{-9}	1.1046834	[1.07-1.14]	*INTS11*	
rs4976646-G	3×10^{-9}	1.0819606	[1.06-1.11]	*RGS14*	
rs8005161-A	3×10^{-9}	1.1371082	[1.09-1.18]	*GPR65*	
rs4656958-G	3×10^{-9}	1.0859073	-	*ITLN2, ITLN1*	
rs1077773-A	6×10^{-9}	1.0747931	-	*LINC02888*	
rs6062504-G	1×10^{-8}	1.0814869	-	*ZGPAT*	
rs11229555-C	1×10^{-8}	1.0858196	-	*GLYAT*	
rs12718244-A	1×10^{-8}	1.0744148	[1.05-1.1]	*SPMIP7*	
rs12149608-?	2×10^{-8}	-	-	*ZFP90*	
rs11230563-G	2×10^{-8}	1.077956	-	*CD6*	
rs11010067-G	2×10^{-8}	1.075212	[1.05-1.1]	*LINC02635, CUL2*	
rs7556897-A	3×10^{-8}	1.0756639	-	*CCL20, SNRPGP8*	
rs11083840-C	3×10^{-8}	1.0715772	[1.05-1.1]	*CALM3, PTGIR*	

Variant and risk allele (rs)	P-value	OR	CI	Gene	References
rs17736589-G	4×10^{-8}	1.0858142	[1.06-1.12]	*CYTH1*	
rs17771967-G	5×10^{-8}	1.073007	[1.05-1.1]	*KIR3DL2, RNU6-222P*	
rs7554511-C	7×10^{-31}	1.1808171	-	*INAVA*	
rs75900472-C	1×10^{-28}	1.1539809	[1.13-1.18]	*HNRNPA1P41, JAK2*	
rs10781499-A	4×10^{-26}	1.142511	[1.12-1.17]	*CARD9*	
rs6920220-A	5×10^{-22}	1.1582799	[1.13-1.19]	*LINC03004*	
rs10761659-G	2×10^{-20}	1.1244732	-	*LINC02929, ALDH7A1P4*	
rs80174646-C	4×10^{-62}	1.614317	-	*IL23R*	
rs4845604-G	1×10^{-18}	1.1778549	-	*RORC*	
rs11742570-G	6×10^{-11}	1.0874969	-	*RNU1-150P, TTC33*	
rs12942547-A	7×10^{-11}	1.0883672	-	*STAT3*	
rs2189234-A	2×10^{-10}	1.0847058	[1.06-1.11]	*TET2*	
rs2111485-A	2×10^{-10}	1.0891383	[1.06-1.12]	*FAP, IFIH1*	
rs13300218-G	2×10^{-10}	1.1441684	-	*NOTCH1*	
rs7240004-A	3×10^{-10}	1.0858526	-	*SMAD7, CTIF*	
rs254562-?	3×10^{-10}	-	-	*PITX1-AS1*	
rs6667605-G	3×10^{-10}	1.0831845	-	*TNFRSF14, PRXL2B*	
rs483905-A	3×10^{-10}	1.0887091	[1.06-1.12]	*MAML2*	
rs7282490-G	2×10^{-15}	1.1100584	[1.09-1.13]	*GATD3*	
rs4246905-G	1×10^{-15}	1.1206568	-	*TNFSF15*	
rs2413583-G	7×10^{-15}	1.1438878	-	*PDGFB, RPL3*	
rs3851228-A	1×10^{-14}	1.2023339	[1.16-1.25]	*TRAF3IP2-AS1*	
rs3749171-A	4×10^{-18}	1.1533448	[1.12-1.19]	*GPR35*	
rs17085007-G	1×10^{-16}	1.1413064	[1.11-1.17]	*RPS21P8, FGFR1OP2P1*	
rs4812833-A	2×10^{-16}	1.1088746	-	*LINC01620*	
rs1182188-A	5×10^{-15}	1.1136187	-	*GNA12*	
rs10185424-A	1×10^{-14}	1.1014192	[1.08-1.13]	*IL1R1, IL1R2*	
rs4728142-A	2×10^{-14}	1.1018242	[1.08-1.13]	*KCP, IRF5*	
rs17229285-G	3×10^{-14}	1.1000017	[1.08-1.12]	*RNU7-147P, PLCL1*	
rs3774937-G	5×10^{-14}	1.1043711	[1.08-1.13]	*NFKB1*	
rs3766606-C	6×10^{-14}	1.143554	-	*PARK7*	
rs941823-G	1×10^{-13}	1.1147928	-	*LINC00598*	
rs1893217-G	3×10^{-13}	1.1283141	[1.1-1.16]	*PTPN2*	
rs9868809-A	3×10^{-13}	1.1570203	[1.12-1.2]	*CELSR3*	
rs12568930-A	4×10^{-13}	1.1360025	-	*PPIAP34, ZBTB40*	
rs17780256-A	6×10^{-13}	1.1223103	-	*SLC39A11*	
rs2823286-G	2×10^{-12}	1.1033705	-	*LINC02920, CYCSP42*	
rs17694108-A	6×10^{-12}	1.1005878	[1.07-1.13]	*SLC7A10, CEBPA*	
rs395157-A	9×10^{-12}	1.0890996	[1.06-1.11]	*OSMR*	
rs10910092-?	1×10^{-11}	-	-	*TNFRSF14, PRXL2B*	
rs12720356-C	2×10^{-11}	1.1656502	[1.12-1.21]	*TYK2*	
rs10010325-?	2×10^{-11}	-	-	*TET2-AS1, TET2*	
rs17622378-G	4×10^{-11}	1.0865855	[1.06-1.11]	*IRF1, CARINH*	
rs1728785-G	3×10^{-8}	1.17	[1.07-1.27]	*ZFP90*	[2]/ [91]
rs886774-G	3×10^{-8}	1.11	[1.03-1.19]	*DLD, PIGCP2*	
rs6017342-C	9×10^{-17}	1.17	[1.09-1.26]	*LINC01620*	
rs11209026-?	3×10^{-10}	-	-	*IL23R*	
rs6426833-?	2×10^{-11}	-	-	*OTUD3, RNF186-AS1*	
rs9858542-?	7×10^{-9}	-	-	*BSN*	

Variant and risk allele (rs)	P-value	OR	CI	Gene	References
rs16940186-G	4×10^{-10}	1.56	[1.36-1.80]	*LINC02132, LINC01082*	[3]/ [88]
rs4654903-?	7×10^{-9}	1.56	[1.35-1.82]	*RNF186-AS1, OTUD3*	
rs16940202-C	3×10^{-9}	1.52	[1.32-1.74]	*LINC01082, LINC02132*	
rs6426833-A	4×10^{-35}	1.3	[1.25-1.35]	*OTUD3, RNF186-AS1*	[4]/ [67]
rs2836878-G	2×10^{-22}	1.25	[1.20-1.32]	*LINC02940, RPL23AP12*	
rs35675666-G	5×10^{-9}	1.08	[1.02-1.15]	*PARK7*	
rs7524102-A	2×10^{-13}	1.1	[1.05-1.16]	*ZBTB40, PPIAP34*	
rs2310173-T	3×10^{-12}	1.09	[1.05-1.14]	*IL1R1, IL1R2*	
rs254560-A	1×10^{-9}	1.07	[1.03-1.12]	*PITX1-AS1*	
rs4246905-C	6×10^{-12}	1.1	[1.05-1.15]	*TNFSF15*	
rs798502-A	3×10^{-15}	1.13	[1.08-1.18]	*GNA12, AMZ1*	
rs11209026-G	5×10^{-28}	1.74	[1.57-1.92]	*IL23R*	
rs4728142-A	2×10^{-8}	1.07	[1.03-1.11]	*KCP, IRF5*	
rs11739663-T	3×10^{-8}	1.15	[1.09-1.21]	*CEP72-DT*	
rs10781499-A	3×10^{-19}	1.12	[1.08-1.17]	*CARD9*	
rs2155219-T	5×10^{-16}	1.13	[1.08-1.17]	*EMSY, LINC02757*	
rs941823-C	4×10^{-12}	1.12	[1.07-1.17]	*LINC00598*	
rs7554511-C	2×10^{-13}	1.19	[1.14-1.25]	*INAVA*	
rs3194051-G	4×10^{-8}	1.07	[1.02-1.12]	*IL7R*	
rs943072-G	2×10^{-10}	1.15	[1.08-1.23]	*LINC02537, VEGFA*	
rs6911490-T	1×10^{-8}	1.08	[1.03-1.13]	*ATG5, PRDM1*	
rs678170-A	5×10^{-14}	1.09	[1.05-1.14]	*NXPE4, NXPE1*	
rs267939-C	6×10^{-12}	1.1	[1.06-1.15]	*DAP*	
rs6451493-T	3×10^{-9}	1.08	[1.04-1.12]	*TTC33, RNU1-150P*	
rs6871626-A	1×10^{-21}	1.17	[1.12-1.22]	*LINC01845, IL12B*	
rs6920220-A	8×10^{-17}	1.14	[1.09-1.20]	*LINC03004*	
rs12261843-G	7×10^{-10}	1.07	[1.03-1.12]	*CCNY, CCNY-AS1*	
rs907611-A	1×10^{-10}	1.08	[1.03-1.13]	*LSP1*	
rs3024505-A	6×10^{-17}	1.25	[1.19-1.32]	*Y_RNA, IL10*	
rs1297265-A	7×10^{-13}	1.11	[1.06-1.15]	*LINC02920, CYCSP42*	
rs17085007-C	1×10^{-16}	1.16	[1.10-1.21]	*RPS21P8, FGFR1OP2P1*	
rs2297441-A	2×10^{-10}	1.09	[1.04-1.15]	*RTEL1, RTEL1-TNFRSF6B*	
rs2838519-G	6×10^{-11}	1.14	[1.05-1.22]	*GATD3*	
rs6499188-A	4×10^{-8}	1.14	[1.09-1.20]	*CDH3*	
rs2872507-A	5×10^{-11}	1.15	[1.10-1.19]	*ZPBP2, GSDMB*	
rs6017342-C	1×10^{-20}	1.2	[1.15-1.26]	*LINC01620*	
rs1801274-A	2×10^{-20}	1.21	[1.16-1.26]	*FCGR2A*	
rs7608910-G	2×10^{-14}	1.19	[1.14-1.24]	*PUS10*	
rs4676406-T	8×10^{-11}	1.14	[1.09-1.18]	*AQP12B, GPR35*	
rs9822268-A	2×10^{-17}	1.21	[1.16-1.26]	*APEH*	
rs10758669-C	2×10^{-25}	1.17	[1.12-1.21]	*HNRNPA1P41, JAK2*	
rs4510766-A	2×10^{-16}	1.2	[1.15-1.26]	*DLD, PIGCP2*	
rs6584283-T	8×10^{-21}	1.21	[1.16-1.26]	*LINC01475*	
rs7134599-A	1×10^{-16}	1.19	[1.19-1.24]	*IFNG-AS1*	
rs734999-C	3×10^{-9}	1.05	[1.01-1.09]	*PRXL2B, TNFRSF14*	
rs11676348-T	1×10^{-10}	1.07	[1.03-1.11]	*CXCR2, CXCR1*	
rs16940202-C	6×10^{-19}	1.15	[1.10-1.21]	*LINC01082, LINC02132*	
rs17207986-G	1×10^{-16}	3.95	[NR]	*TNXB*	[5]/ [73]
rs1801274-?	2×10^{-12}	1.59	[1.39-1.82]	*FCGR2A*	[6]/ [68]

Variant and risk allele (rs)	P-value	OR	CI	Gene	References
rs9263739-T	4×10^{-67}	2.73	[2.43-3.07]	*CCHCR1*	
rs4654925-G	9×10^{-22}	1.41	[1.30-1.54]	*OTUD3*	[7]/ [71]
rs7809799-G	9×10^{-11}	1.56	[1.36-1.78]	*SMURF1, KPNA7*	
rs5771069-G	4×10^{-8}	1.17	[1.11-1.25]	*IL17REL*	
rs3024493-T	1×10^{-12}	1.35	[1.22-1.50]	*IL10*	
rs1558744-A	3×10^{-12}	1.35	-	*IFNG-AS1*	[8]/ [86]
rs11209026-?	1×10^{-8}	1.79	-	*IL23R*	
rs6426833-G	5×10^{-13}	1.37	-	*OTUD3, RNF186-AS1*	
rs3806308-?	7×10^{-9}	1.28	-	*RNF186-AS1*	
rs10889677-A	1×10^{-8}	1.29	-	*IL23R*	
rs3774959-A	4×10^{-12}	1.118	[1.077-1.159]	*NFKB1*	[9]/ [75]
rs798502-A	6×10^{-17}	1.127	[1.084-1.171]	*GNA12, AMZ1*	
rs4380874-T	2×10^{-26}	1.137	[1.097-1.177]	*DLD, PIGCP2*	
rs28374715-A	2×10^{-8}	1.082	[1.04-1.126]	*CHP1*	
rs1728785-C	4×10^{-8}	1.075	[1.031-1.121]	*ZFP90*	
rs6088765-G	2×10^{-8}	1.079	[1.041-1.117]	*MMP24OS, PROCR*	
rs6017342-C	1×10^{-43}	1.228	[1.185-1.273]	*LINC01620*	
rs17229285-C	2×10^{-13}	1.117	[1.079-1.157]	*RNU7-147P, PLCL1*	
rs9847710-C	1×10^{-8}	1.064	[1.027-1.102]	*SFMBT1*	
rs11739663-T	2×10^{-8}	1.071	[1.027-1.117]	*CEP72-DT*	
rs254560-A	3×10^{-9}	1.056	[1.019-1.093]	*PITX1-AS1*	
rs4722672-C	2×10^{-8}	1.091	[1.043-1.14]	*HOXA11-AS, HOXA13*	
rs4728142-A	4×10^{-14}	1.104	[1.066-1.143]	*KCP, IRF5*	
rs483905-A	1×10^{-8}	1.056	[1.017-1.096]	*MAML2*	
rs561722-C	5×10^{-17}	1.12	[1.079-1.163]	*NXPE2P1, NXPE1*	
rs11150589-T	6×10^{-10}	1.09	[1.052-1.128]	*Y_RNA*	
rs7210086-A	2×10^{-9}	1.111	[1.062-1.163]	*LINC00511, SLC39A11*	
rs1126510-G	2×10^{-9}	1.075	[1.037-1.113]	*PTGIR*	
rs10797432-C	3×10^{-12}	1.078	[1.041-1.116]	*PRXL2B, TNFRSF14*	
rs6426833-A	2×10^{-68}	1.265	[1.221-1.31]	*OTUD3, RNF186-AS1*	
rs2816958-G	2×10^{-17}	1.23	[1.161-1.302]	*NR5A2*	
rs1016883-G	3×10^{-8}	1.1	[1.051-1.15]	*PLCL1*	
rs3806308-G	3×10^{-8}	1.15	-	*RNF186-AS1*	[10]/ [83]
rs2836878-G	1×10^{-8}	1.27	[NR]	*LINC02940, RPL23AP12*	
rs10800309-A	3×10^{-9}	1.23	[NR]	*FCGR2A, RNU6-481P*	
rs11190140-T	1×10^{-8}	1.23	[NR]	*LINC01475*	
rs13003464-G	7×10^{-9}	1.18	[NR]	*PUS10*	
rs1317209-T	2×10^{-10}	1.34	[NR]	*RNF186, TMCO4*	
rs1558744-A	4×10^{-12}	1.2	[NR]	*IFNG-AS1*	
rs2201841-C	1×10^{-13}	1.16	[NR]	*IL23R, C1orf141*	
rs2305480-T	3×10^{-8}	1.25	[NR]	*GSDMB*	
rs3024505-T	1×10^{-8}	1.28	[NR]	*Y_RNA, IL10*	
rs3197999-T	4×10^{-9}	1.21	[NR]	*MST1*	
rs4077515-A	5×10^{-8}	1.22	[NR]	*CARD9*	
rs4957048-C	1×10^{-9}	1.11	[NR]	*CEP72-DT*	
rs6426833-A	2×10^{-21}	1.3	[NR]	*OTUD3, RNF186-AS1*	
rs3024505-T	1×10^{-12}	1.46	[1.31-1.62]	*Y_RNA, IL10*	[11]/ [70]
rs2858829-?	8×10^{-10}	1.12	[1.08-1.16]	*CBX3P9, DSE*	[12]/ [76]
rs1049526-?	2×10^{-8}	2.3810558	[1.74-3.26]	*BRD2*	[13]/ [84]

Variant and risk allele (rs)	P-value	OR	CI	Gene	References
rs501916-?	4 x 10^{-10}	1.8129687	[1.51-2.18]	*SEMA6D*	
rs1811711-C	6 x 10^{-9}	1.14	[1.10–1.18]	*CCL20*	[14]/ [69]
rs17656349-T	2 x 10^{-8}	1.09	[1.06–1.13]	*CAMK2A*	
rs113986290-C	8 x 10^{-9}	1.36	[1.25–1.46]	*LNC-LBCS*	
rs7911117-T	2 x 10^{-8}	1.14	[1.10–1.19]	*FAM238C, ABI1*	
rs138788-A	3 x 10^{-8}	1.09	[1.06–1.13]	*TOM1*	
rs6426833-?	3 x 10^{-42}	-	-	*OTUD3, RNF186-AS1*	
rs11581607-?	4 x 10^{-41}	-	-	*IL23R*	
rs2836878-?	2 x 10^{-32}	-	-	*LINC02940, RPL23AP12*	
rs6017342-?	4 x 10^{-30}	-	-	*LINC01620*	
rs11614178-?	1 x 10^{-29}	-	-	*IFNG-AS1*	
rs56167332-?	1 x 10^{-23}	-	-	*IL12B, LINC01845*	
rs3024505-?	2 x 10^{-23}	-	-	*Y_RNA, IL10*	
rs7608910-?	4 x 10^{-23}	-	-	*PUS10*	
rs4409764-?	2 x 10^{-21}	-	-	*LINC01475*	
rs4380874-?	9 x 10^{-21}	-	-	*DLD, PIGCP2*	
rs3197999-?	8 x 10^{-20}	-	-	*MST1*	
rs75900472-?	1 x 10^{-19}	-	-	*HNRNPA1P41, JAK2*	
rs1801274-?	2 x 10^{-18}	-	-	*FCGR2A*	
rs4676408-?	1 x 10^{-17}	-	-	*GPR35, AQP12B*	
rs6062496-?	9 x 10^{-17}	-	-	*TNFRSF6B, RTEL1-TNFRSF6B*	
rs12946510-?	2 x 10^{-16}	-	-	*IKZF3, GRB7*	
rs10781499-?	2 x 10^{-16}	-	-	*CARD9*	
rs7554511-?	4 x 10^{-16}	-	-	*INAVA*	
rs10761659-?	1 x 10^{-15}	-	-	*LINC02929, ALDH7A1P4*	
rs12568930-?	2 x 10^{-15}	-	-	*PPIAP34, ZBTB40*	
rs6920220-?	3 x 10^{-15}	-	-	*LINC03004*	
rs11236797-?	5 x 10^{-15}	-	-	*LINC02757, EMSY*	
rs17085007-?	1 x 10^{-14}	-	-	*RPS21P8, FGFR1OP2P1*	
rs2823286-?	2 x 10^{-13}	-	-	*LINC02920, CYCSP42*	
rs2816958-?	2 x 10^{-13}	-	-	*NR5A2*	
rs78534766-?	3 x 10^{-13}	-	-	*ADCY7*	
rs7282490-?	5 x 10^{-13}	-	-	*GATD3*	
rs4845604-?	2 x 10^{-11}	-	-	*RORC*	
rs798502-?	4 x 10^{-11}	-	-	*GNA12, AMZ1*	
rs12942547-?	1 x 10^{-10}	-	-	*STAT3*	
rs10797432-?	1 x 10^{-10}	-	-	*PRXL2B, TNFRSF14*	
rs5771069-?	2 x 10^{-10}	-	-	*IL17REL*	
rs16940202-?	3 x 10^{-10}	-	-	*LINC01082, LINC02132*	
rs2413583-?	3 x 10^{-10}	-	-	*PDGFB, RPL3*	
rs4728142-?	3 x 10^{-10}	-	-	*KCP, IRF5*	
rs17780256-?	4 x 10^{-10}	-	-	*SLC39A11*	
rs3851228-?	6 x 10^{-10}	-	-	*TRAF3IP2-AS1*	
rs200349593-?	2 x 10^{-9}	-	-	*NXPE1*	
rs3766606-?	4 x 10^{-9}	-	-	*PARK7*	
rs17694108-?	1 x 10^{-8}	-	-	*SLC7A10, CEBPA*	
rs17293632-?	2 x 10^{-8}	-	-	*SMAD3*	
rs254560-?	3 x 10^{-8}	-	-	*PITX1-AS1*	
rs1728785-?	4 x 10^{-8}	-	-	*ZFP90*	

Variant and risk allele (rs)	P-value	OR	CI	Gene	References
rs4976646-?	4×10^{-8}	-	-	*RGS14*	
rs76418789-?	9×10^{-11}	1.9607843	[1.59-2.38]	*C1orf141, IL23R*	
rs549182-A	8×10^{-10}	1.59	[1.37-1.85]	*TSBP1-AS1, NOTCH4*	[15]/ [77]
rs4151657-G	5×10^{-14}	1.54	[1.38-1.73]	*CFB*	
rs3749946-A	2×10^{-15}	1.97	[1.66-2.33]	*HCP5, MICB-DT*	
rs1830610-T	2×10^{-9}	1.35	-	*RLN2, INSL4*	[16]/ [89]
rs2269426-A	4×10^{-11}	0.872	[0.837-0.908]	*TNXB*	[17]/ [78]
rs3823418-A	4×10^{-8}	0.858	[0.813-0.906]	*PSORS1C1*	
rs2523971-A	1×10^{-9}	0.874	[0.836-0.913]	*POLR1HASP*	
rs62037369-A	2×10^{-8}	1.12	[1.077-1.165]	*SH2B1*	
rs2844782-A	1×10^{-8}	1.128	[1.082-1.176]	*TRIM15, TRIM26*	
rs2844458-A	5×10^{-8}	1.118	[1.074-1.164]	*EHMT2, EHMT2-AS1*	
rs6088728-A	3×10^{-8}	1.118	[1.075-1.162]	*EDEM2*	
rs2269426-A	2×10^{-8}	0.922	[0.896-0.948]	*TNXB*	
rs10799837-A	7×10^{-12}	-	[0.15-0.27]	*RNF186, TMCO4*	[18]/ [81]
rs145568234-G	1×10^{-26}	-	[0.72-1.04]	*TSBP1-AS1*	
rs2836882-A	1×10^{-13}	-	[0.19-0.33]	*LINC02940, RPL23AP12*	
rs4654925-C	8×10^{-13}	-	[0.16-0.27]	*OTUD3*	
rs6017342-?	5×10^{-46}	-	[0.16-0.21]	*LINC01620*	[19]/ [80]
rs6062496-?	4×10^{-26}	-	[0.11-0.16]	*TNFRSF6B, RTEL1-TNFRSF6B*	
rs7277261-?	4×10^{-18}	-	[0.092-0.145]	*LINC02920, CYCSP42*	
rs2836882-?	2×10^{-45}	-	[0.17-0.22]	*LINC02940, RPL23AP12*	
rs12158299-?	5×10^{-14}	-	[0.072-0.122]	*UBE2L3, YDJC*	
rs1003342-?	2×10^{-9}	-	[0.047-0.093]	*HORMAD2*	
rs4821382-?	3×10^{-10}	-	[0.058-0.11]	*RNU7-167P, LINC01399*	
rs9611131-?	5×10^{-21}	-	[0.14-0.21]	*PDGFB, RPL3*	
rs5771192-?	1×10^{-17}	-	[0.078-0.125]	*IL17REL*	
rs10799591-?	5×10^{-24}	0.74282116	-	*RNF186-AS1, OTUD3*	
rs10493860-?	2×10^{-8}	0.85770077	-	*TGFBR3*	
rs6671847-?	3×10^{-11}	0.82365793	-	*FCGR2A*	
rs10010281-?	5×10^{-8}	0.87494	-	*TET2-AS1, TET2*	
rs4712651-?	3×10^{-11}	0.8259674	-	*CASC15*	
rs10974900-?	4×10^{-12}	0.83979285	-	*JAK2*	
rs10882847-?	8×10^{-10}	1.2119129	-	*LCOR*	
rs17085007-?	4×10^{-25}	1.330294	-	*RPS21P8, FGFR1OP2P1*	
rs201121732-?	2×10^{-8}	1.6293807	-	*IL4R, IL21R*	
rs34372308-?	4×10^{-10}	0.85547364	-	*MIEN1, GRB7*	
rs2836884-?	4×10^{-10}	0.82712454	-	*LINC02940, RPL23AP12*	
rs9726836-?	4×10^{-9}	-	[0.062-0.124]	*CCNL2*	
rs1886730-?	6×10^{-16}	-	[0.071-0.116]	*TNFRSF14*	
rs225131-?	9×10^{-18}	-	[0.09-0.143]	*ERRFI1-DT*	
rs6426833-?	1×10^{-74}	-	[0.19-0.24]	*OTUD3, RNF186-AS1*	
rs34963268-?	1×10^{-29}	-	[0.15-0.21]	*ZBTB40, PPIAP34*	
rs12138864-?	4×10^{-8}	-	[0.041-0.088]	*PHC2*	
rs6687307-?	5×10^{-8}	-	[0.055-0.116]	*LINC01767, PLPP3*	
rs11581607-?	8×10^{-49}	-	[0.39-0.51]	*IL23R*	
rs7552319-?	7×10^{-9}	-	[0.048-0.097]	*CASP3P1, CHORDC1P5*	
rs1336900-?	5×10^{-10}	-	[0.052-0.099]	*HORMAD1*	
rs11204894-?	2×10^{-12}	-	[0.073-0.13]	*RORC*	

Variant and risk allele (rs)	P-value	OR	CI	Gene	References
rs6658353-?	3 x 10^{-39}	-	[0.14-0.18]	*RNU6-481P, FCGR2A*	
rs2816980-?	3 x 10^{-12}	-	[0.11-0.19]	*NR5A2*	
rs35730213-?	2 x 10^{-24}	-	[0.12-0.18]	*INAVA*	
rs11240504-?	2 x 10^{-8}	-	[0.048-0.1]	*CDK18*	
rs3024495-?	2 x 10^{-45}	-	[0.2-0.26]	*IL10*	
rs11582528-?	2 x 10^{-8}	-	[0.056-0.116]	*PSEN2, RPS27P5*	
rs10910476-?	3 x 10^{-8}	-	[0.043-0.091]	*U8, IRF2BP2*	
rs7578575-?	4 x 10^{-11}	-	[0.059-0.109]	*DNMT3A*	
rs67927699-?	2 x 10^{-28}	-	[0.12-0.17]	*PUS10*	
rs1882348-?	5 x 10^{-15}	-	[0.068-0.114]	*IL18R1*	
rs72837826-?	1 x 10^{-8}	-	[0.078-0.16]	*MIR4435-2HG*	
rs2124440-?	1 x 10^{-8}	-	[0.045-0.093]	*ITGA4*	
rs34119476-?	2 x 10^{-19}	-	[0.082-0.127]	*RNU7-147P, PLCL1*	
rs3769684-?	6 x 10^{-10}	-	[0.078-0.149]	*CD28*	
rs11690316-?	7 x 10^{-13}	-	[0.06-0.106]	*CXCR1, CXCR2*	
rs4973341-?	3 x 10^{-8}	-	[0.046-0.097]	*CCL20, SNRPGP8*	
rs34236350-?	9 x 10^{-33}	-	[0.14-0.19]	*GPR35*	
rs113010081-?	2 x 10^{-11}	-	[0.095-0.173]	*LINC02009, CCRL2*	
rs1131095-?	1 x 10^{-36}	-	[0.14-0.19]	*APEH*	
rs138317798-?	1 x 10^{-8}	-	[0.088-0.182]	*RPL29, DUSP7*	
rs56116661-?	3 x 10^{-8}	-	[0.057-0.12]	*LPP*	
rs2903386-?	6 x 10^{-13}	-	[0.062-0.108]	*TET2, TET2-AS1*	
rs147909357-?	2 x 10^{-9}	-	[0.047-0.093]	*IL21, IL2*	
rs4081846-?	2 x 10^{-8}	-	[0.072-0.15]	*CEP72-DT*	
rs3776414-?	3 x 10^{-8}	-	[0.044-0.091]	*DAP*	
rs395157-?	2 x 10^{-10}	-	[0.051-0.097]	*OSMR*	
rs2188962-?	2 x 10^{-8}	-	[0.049-0.1]	*CARINH*	
rs254560-?	2 x 10^{-13}	-	[0.064-0.111]	*PITX1-AS1*	
rs1000113-?	6 x 10^{-18}	-	[0.11-0.18]	*IRGM*	
rs755374-?	6 x 10^{-34}	-	[0.12-0.17]	*IL12B, LINC01845*	
rs564349-?	4 x 10^{-10}	-	[0.054-0.103]	*ERGIC1*	
rs10051765-?	4 x 10^{-10}	-	[0.053-0.101]	*RGS14, SLC34A1*	
rs55982276-?	9 x 10^{-9}	-	[0.095-0.193]	*PHYKPL*	
rs17119-?	6 x 10^{-9}	-	[0.063-0.127]	*RNU6-793P, RPL6P17*	
rs181635615-?	9 x 10^{-9}	-	[0.2-0.41]	*LNC-LBCS*	
rs2328546-?	1 x 10^{-10}	-	[0.063-0.118]	*CDKAL1*	
rs4710973-?	6 x 10^{-10}	-	[0.052-0.1]	*LINC00581, CDKAL1*	
rs9480634-?	2 x 10^{-8}	-	[0.044-0.092]	*PRDM1*	
rs73534586-?	1 x 10^{-13}	-	[0.14-0.25]	*TRAF3IP2-AS1, TRAF3IP2*	
rs2858829-?	9 x 10^{-9}	-	[0.044-0.089]	*CBX3P9, DSE*	
rs6933404-?	8 x 10^{-19}	-	[0.11-0.17]	*BTF3L4P3, LINC03004*	
rs2260230-?	1 x 10^{-16}	-	[0.081-0.131]	*GNA12*	
rs748670681-?	6 x 10^{-61}	-	[0.69-0.88]	*TNRC18*	
rs1990134-?	1 x 10^{-9}	-	[0.052-0.102]	*GLI3, INHBA-AS1*	
rs6976037-?	5 x 10^{-9}	-	[0.046-0.093]	*EPS15P1, MRPL42P4*	
rs7898978-?	1 x 10^{-8}	-	[0.046-0.095]	*CCND3P1, MAP3K8*	
rs12764283-?	4 x 10^{-10}	-	[0.053-0.101]	*CCNY-AS1*	
rs10761659-?	3 x 10^{-18}	-	[0.08-0.126]	*LINC02929, ALDH7A1P4*	
rs11203002-?	3 x 10^{-12}	-	[0.083-0.147]	*MIR4679-2, CH25H*	

Variant and risk allele (rs)	P-value	OR	CI	Gene	References
rs7918084-?	1×10^{-9}	-	[0.051-0.099]	*EIF2S2P3*	
rs11188950-?	3×10^{-11}	-	[0.11-0.21]	*LCOR*	
rs4409764-?	3×10^{-40}	-	[0.13-0.17]	*LINC01475*	
rs113369211-?	2×10^{-8}	-	[0.13-0.27]	*BTRC*	
rs11591780-?	5×10^{-9}	-	[0.046-0.091]	*ACTR1A, MFSD13A*	
rs3757387-?	2×10^{-17}	-	[0.08-0.128]	*KCP, IRF5*	
rs77535993-?	6×10^{-9}	-	[0.069-0.139]	*IDI1P2, UBE2V2*	
rs56211063-?	5×10^{-12}	-	[0.1-0.19]	*DELEC1, TNFSF15*	
rs4380874-?	2×10^{-28}	-	[0.11-0.16]	*DLD, PIGCP2*	
rs2721933-?	2×10^{-8}	-	[0.046-0.095]	*TRPS1*	
rs36051895-?	9×10^{-34}	-	[0.12-0.17]	*JAK2, HNRNPA1P41*	
rs3812565-?	2×10^{-18}	-	[0.082-0.129]	*SNAPC4*	
rs2790211-?	2×10^{-9}	-	[0.056-0.11]	*IPMK*	
rs907613-?	2×10^{-11}	-	[0.06-0.109]	*LSP1*	
rs10896794-?	2×10^{-8}	-	[0.058-0.121]	*LPXN*	
rs71040008-?	2×10^{-22}	-	[0.1-0.15]	*EMSY, LINC02757*	
rs561722-?	9×10^{-15}	-	[0.069-0.116]	*NXPE2P1, NXPE1*	
rs1558746-?	1×10^{-32}	-	[0.16-0.22]	*IFNG-AS1*	
rs11066188-?	2×10^{-8}	-	[0.049-0.102]	*HECTD4*	
rs17085007-?	4×10^{-35}	-	[0.15-0.2]	*RPS21P8, FGFR1OP2P1*	
rs17630801-?	7×10^{-10}	-	[0.11-0.21]	*ELF1*	
rs4902367-?	3×10^{-9}	-	[0.051-0.102]	*RNU2-14P, LINC02324*	
rs4462528-?	1×10^{-13}	-	[0.099-0.17]	*GALC*	
rs56375023-?	2×10^{-10}	-	[0.064-0.122]	*SMAD3*	
rs71407313-?	2×10^{-11}	-	[0.097-0.176]	*CRTC3*	
rs11641184-?	3×10^{-11}	-	[0.054-0.1]	*LITAF*	
rs59790099-?	2×10^{-11}	-	[0.066-0.12]	*PRKCB*	
rs201121732-?	3×10^{-8}	-	[0.15-0.3]	*IL4R, IL21R*	
rs77948921-?	9×10^{-12}	-	[0.062-0.112]	*SMG1P5*	
rs6499186-?	3×10^{-9}	-	[0.059-0.118]	*ZFP90, CDH3*	
rs11548656-?	4×10^{-8}	-	[0.12-0.26]	*PLCG2*	
rs16940202-?	1×10^{-27}	-	[0.13-0.19]	*LINC01082, LINC02132*	
rs4795894-?	5×10^{-8}	-	[0.044-0.093]	*CCL7, CCL2*	
rs11658993-?	1×10^{-32}	-	[0.11-0.16]	*IKZF3*	
rs9911533-?	2×10^{-14}	-	[0.069-0.116]	*CCR7, SMARCE1*	
rs12601611-?	5×10^{-20}	-	[0.091-0.14]	*STAT3*	
rs8072795-?	2×10^{-10}	-	[0.084-0.159]	*LINC00511, SLC39A11*	
rs116885423-?	2×10^{-8}	-	[0.29-0.61]	*CEP131, PVALEF*	
rs547268-?	2×10^{-10}	-	[0.063-0.118]	*PTPN2*	
rs12720356-?	5×10^{-10}	-	[0.096-0.185]	*TYK2*	
rs138636798-?	2×10^{-16}	-	[0.1-0.17]	*CEBPA, SLC7A10*	
rs11672983-?	2×10^{-9}	-	[0.049-0.096]	*RNU6-222P, FCAR*	
rs74365910-A	9×10^{-10}	-	[0.66-1.28]	*PSMB8, TAP2*	[20]/ [74]
rs115378818-C	2×10^{-29}	-	[0.75-0.87]	*TSBP1-AS1, TSBP1*	[21]/ [87]
rs147219747-A	1×10^{-11}	-	[1.64-1.78]	*CEP128*	
rs11581607-G	2×10^{-12}	-	[0.27-0.47]	*IL23R*	
rs182307779-A	6×10^{-13}	-	[3.18-3.23]	*ATXN10*	
rs111771292-A	8×10^{-12}	-	[2.31-2.4]	*KIAA0319*	
rs148844907-T	3×10^{-12}	-	[0.72-0.91]	*C6orf47-AS1, C6orf47*	

Variant and risk allele (rs)	P-value	OR	CI	Gene	References
rs115378818-C	5 x 10^{-20}	-	[0.85-0.99]	*TSBP1-AS1, TSBP1*	
rs1887428-G	1 x 10^{-11}	-	[0.094-0.184]	*JAK2*	
rs559689141-C	2 x 10^{-12}	-	[2.95-3]	*STK24-AS1, NUS1P4*	
rs6426833-G	5 x 10^{-13}	-	[0.1-0.16]	*OTUD3, RNF186-AS1*	
rs113155999-T	9 x 10^{-14}	-	[0.23-0.48]	*DNAJB6P4, RNU4ATAC4P*	
rs10800314-C	2 x 10^{-13}	-	[0.098-0.182]	*RNU6-481P, FCGR2A*	
rs115378818-C	7 x 10^{-53}	-	[0.88-0.96]	*TSBP1-AS1, TSBP1*	
rs148897986-C	6 x 10^{-12}	-	[2.62-2.7]	*TSEN15P3, LINC03007*	
rs9977672-G	6 x 10^{-12}	-	[0.1-0.2]	*RPL23AP12, LINC02940*	
rs567689672-A	2 x 10^{-11}	-	[3.23-3.28]	*SEPTIN9*	
rs140010323-C	7 x 10^{-12}	-	[2.18-2.28]	*YES1*	
rs545789536-C	6 x 10^{-12}	-	[2.04-2.15]	*ANKRD12*	
rs577185043-A	2 x 10^{-11}	-	[3.09-3.15]	*BTBD3, PA2G4P2*	
chr1:20227723-G	9 x 10^{-13}	-	[0.1-0.18]	-	
chr1:67722919-C	4 x 10^{-15}	-	[0.28-0.47]	-	
rs10800314-C	3 x 10^{-13}	-	[0.11-0.19]	*RNU6-481P, FCGR2A*	
rs1887428-G	7 x 10^{-13}	-	[0.11-0.19]	*JAK2*	
rs78534766-?	1 x 10^{-14}	2.16	[1.77-2.62]	*ADCY7*	[22]/ [82]
rs748670681-T	2 x 10^{-16}	-	[0.56-0.91]	*TNRC18*	[23]/ [85]
rs10748781-A	5 x 10^{-10}	-	[0.085-0.163]	*LINC01475*	
rs528766038-T	6 x 10^{-9}	-	[0.23-0.46]	*SLIT1*	
rs7936434-C	6 x 10^{-9}	-	[0.077-0.155]	*EMSY, LINC02757*	
rs7134599-A	4 x 10^{-8}	-	[0.073-0.153]	*IFNG-AS1*	
rs4065985-C	1 x 10^{-8}	-	[0.071-0.147]	*LRRC3C, GSDMA*	
rs6017342-C	7 x 10^{-11}	-	[0.09-0.167]	*LINC01620*	
rs4817983-C	6 x 10^{-14}	-	[0.13-0.22]	*RPL23AP12, LINC02940*	
rs1521186-A	1 x 10^{-9}	-	[0.08-0.157]	*RORC*	
rs1801274-G	2 x 10^{-16}	-	[0.12-0.2]	*FCGR2A*	
rs113307843-AC	2 x 10^{-9}	-	[0.093-0.184]	-	
rs10737481-G	5 x 10^{-18}	-	[0.13-0.21]	*RNF186-AS1, OTUD3*	
rs3024493-A	1 x 10^{-11}	-	[0.13-0.24]	*IL10*	
rs79755370-A	2 x 10^{-11}	-	[0.22-0.4]	*IL23R*	
rs34236350-T	2 x 10^{-10}	-	[0.1-0.19]	*GPR35*	
rs2278300-G	1 x 10^{-9}	-	[0.085-0.165]	*PUS10*	
rs2918392-C	1 x 10^{-8}	-	[0.077-0.157]	*DAP*	
rs145568234-G	5 x 10^{-35}	-	[0.99-1.37]	*TSBP1-AS1*	
rs990107-G	9 x 10^{-10}	-	[0.082-0.159]	*PIGCP2, DLD*	
rs6426833-G	7 x 10^{-12}	0.85	-	*OTUD3, RNF186-AS1*	[24]/ [90]
rs11581607-A	7 x 10^{-11}	0.7	-	*IL23R*	
rs2836878-A	2 x 10^{-16}	0.8	-	*LINC02940, RPL23AP12*	
rs6017342-A	2 x 10^{-9}	0.87	-	*LINC01620*	
rs1801274-A	1 x 10^{-11}	1.17	-	*FCGR2A*	
rs6920220-A	5 x 10^{-8}	1.16	-	*LINC03004*	
rs2816958-A	1 x 10^{-9}	0.78	-	*NR5A2*	
rs41291790-A	3 x 10^{-8}	5.3	-	*NCR3, UQCRHP1*	[25]/ [72]

Sample size and population in GWAS studies

[1]6,968 European ancestry cases, 20,464 European ancestry controls

[2]2,361 European ancestry cases, 5,417 European ancestry controls

[3]388 Korean ancestry cases, 739 Korean ancestry controls

[4]6,687 European ancestry cases, 19,718 European ancestry controls
[5]324 European ancestry medically refractory ulcerative colitis cases, 537 European ancestry non-medically refractory ulcerative colitis cases, 2,601 European ancestry controls
[6]376 Japanese ancestry cases, 934 Japanese ancestry controls
[7]1,043 European ancestry cases, 1,703 European ancestry controls
[8]1,022 European ancestry cases, 2,503 European ancestry controls
[9] Up to 12,924 European ancestry cases, up to 21,442 European ancestry controls
[10] 2,693 European ancestry cases, 6,791 European ancestry controls
[11] 825 patients and 1525 healthy controls from Spain
[12] up to 7,483 European ancestry cases, up to 21,211 European ancestry controls
[13] 269 Polish ancestry child cases, 259 Polish ancestry adult cases, 582 Polish ancestry controls
[14] 12,366 European and unknown ancestry cases, 33,609 European and unknown ancestry controls
[15] 603 North Indian ancestry cases, 622 North Indian ancestry controls
[16] 705 Korean ancestry cases, 1,178 Korean ancestry controls
[17] 14,723 European ancestry cases, 39,125 European ancestry controls
[18] 394,626 European ancestry individuals
[19] 6,862 East Asian ancestry cases, 15,456 East Asian ancestry controls, 16,390 European ancestry cases, 336,800 European ancestry controls
[20] 2,569 European ancestry cases, 453,779 European ancestry controls
[21] 513 African American or Afro-Caribbean cases, 54,992 African American or Afro-Caribbean controls, 4,252 European ancestry cases, 311,416 European ancestry controls, 338 Hispanic or Latin American cases, 28,976 Hispanic or Latin American controls
[22] 7,760 European ancestry cases, 16,065 European ancestry controls
[23] 5,371 European ancestry cases, 412,561 European ancestry controls, 314 East Asian ancestry cases, 178,375 East Asian ancestry controls
[24] 5,371 European ancestry cases, 412,561 European ancestry controls, 314 East Asian ancestry cases, 178,375 East Asian ancestry controls
[25] 208 European ancestry cases, 935 European ancestry controls
